# Sensitivity of Five Rapid HIV Tests on Oral Fluid or Finger-Stick Whole Blood: A Real-Time Comparison in a Healthcare Setting

**DOI:** 10.1371/journal.pone.0011581

**Published:** 2010-07-19

**Authors:** Juliette Pavie, Anne Rachline, Bénédicte Loze, Laurence Niedbalski, Constance Delaugerre, Eric Laforgerie, Jean-Christophe Plantier, Willy Rozenbaum, Sylvie Chevret, Jean-Michel Molina, François Simon

**Affiliations:** 1 Service des Maladies Infectieuses et Tropicales, Hôpital Saint-Louis, Université Paris Diderot, Paris, France; 2 Service de Microbiologie, Hôpital Saint-Louis, Université Paris Diderot, Paris, France; 3 Agence Française de Sécurité Sanitaire et des Produits de Santé, Saint Denis, France; 4 Laboratoire Associé du Centre National de Référence sur le VIH, CHU Charles Nicolle, Rouen, France; 5 Département de Biostatistique et Informatique Médicale, Hôpital Saint-Louis, Université Paris Diderot, Paris, France; McGill University Health Center, Canada

## Abstract

**Background:**

Health authorities in several countries recently recommended the expansion of human immunodeficiency virus (HIV) antibody testing, including the use of rapid tests. Several HIV rapid tests are now licensed in Europe but their sensitivity on total blood and/or oral fluid in routine healthcare settings is not known.

**Methods and Findings:**

200 adults with documented HIV-1 (n = 194) or HIV-2 infection (n = 6) were prospectively screened with five HIV rapid tests using either oral fluid (OF) or finger-stick whole blood (FSB). The OraQuick Advance rapid HIV1/2® was first applied to OF and then to FSB, while the other tests were applied to FSB, in the following order: Vikia HIV 1/2®, Determine HIV 1–2®, Determine® HIV-1/2 Ag/Ab Combo® and INSTI HIV-1/HIV-2®. Tests negative on FSB were repeated on paired serum samples. Twenty randomly selected HIV-seronegative subjects served as controls, and the results were read blindly. Most patients had HIV-1 subtype B infection (63.3%) and most were on antiretroviral therapy (68.5%). Sensitivity was 86.5%, 94.5%, 98.5%, 94.9%, 95.8% and 99% respectively, with OraQuick OF, OraQuick FSB, Vikia, Determine, Determine Ag/Ab Combo and INSTI (p<0.0001). OraQuick was less sensitive on OF than on FSB (p = 0.008). Among the six patients with three or more negative tests, two had recent HIV infection and four patients on antiretroviral therapy had undetectable plasma viral load. When patients positive in all the tests were compared with patients who had at least one negative test, only a plasma HIV RNA level <200 cp/ml was significantly associated with a false-negative result (p = 0.009). When the 33 rapid tests negative on FSB were repeated on serum, all but six (5 negative with OraQuick, 1 with INSTI) were positive. The sensitivity of OraQuick, Determine and Determine Ag/Ab Combo was significantly better on serum than on FSB (97.5%, p = 0.04; 100%, p = 0.004; and 100%, p = 0.02, respectively).

**Conclusion:**

When evaluated in a healthcare setting, rapid HIV tests were less sensitive on oral fluid than on finger-stick whole blood and less sensitive on finger-stick whole blood than on serum.

## Introduction

Late diagnosis of human immunodeficiency virus (HIV) infection, resulting in delayed patient management, is associated with poorer survival [1]. About one-third of new diagnoses in industrialized countries are made when the patient is already severely immunosuppressed [2], [3], while in developing countries more than 80% of patients are diagnosed at an advanced clinical stage [4], [5]. In the United States, the Centers for Diseases Control and Prevention have recommended extending HIV antibody testing to people aged 13–64 years [6]. Such a program would be implemented in a variety of healthcare settings, such as hospital emergency departments, and could involve disposable rapid HIV diagnostic tests, the patient receiving the necessary information at the same site [6]. Such HIV rapid tests use finger-stick capillary whole blood (FSB) or oral fluid (OF), thus avoiding the need for venous blood sampling and centrifugation.

Medical laboratories have been using these rapid tests for more than two decades to test serum and plasma, particularly in developing countries and for emergency diagnosis [7]. They are simple to use but lack sensitivity relative to reference enzyme immunoassays (EIA), particularly during primary HIV infection and infection by variant strains [8].

In the EU, these tests must first undergo validation studies of sensitivity and specificity against panels of frozen sera or plasma collected during primary infection and covering the principal HIV variants, previously tested with reference EIA and Western blot methods [9], [10]. Sensitivity testing of rapid tests on whole blood and oral fluid is hindered by the need to test fresh samples and by the lack of a reference panel. No real-time comparisons of such HIV tests are available. Following recent French recommendations to extend HIV testing [11], including the use of rapid testing when necessary, the French agency for health product safety (Afssaps) mandated us to carry out a real-time comparison of the sensitivity of the five approved rapid tests on samples from patients with documented HIV infection.

## Materials and Methods

Two hundred consecutive adults with documented HIV infection and 20 HIV-seronegative volunteers, included to permit blinded test reading, were prospectively recruited in our outpatient clinic in Saint Louis Hospital, Paris, France, from December 2008 to February 2009, with their written informed consent. The study was approved by the Paris-Saint-Louis ethics committee and the Afssaps scientific board.

HIV-1 or HIV-2 infection had previously been confirmed by western blot positivity. (Biorad, New Lav blot, Paris, France). The patients' characteristics (age, sex, CDC stage, geographic origin, HBV and HCV serostatus, antiretroviral therapy, date of HIV infection, CD4 cell count) and the HIV-1 subtype were obtained from our computerized database. Plasma viral load was determined by using the Cobas TaqMan® (Roche V1.0, Meylan, France) or Abbott Real*Time*® method (Abbott Molecular, Rungis, France), and the viral genotype was determined in patients infected with HIV-1 group M by polymerase gene sequencing (ViroSeq®, Celera-Abbott). When viral load was undetectable or the patient was infected by a variant, serotyping was used to differentiate between subtype B and non B anti-V3 antibodies, and between anti-HIV-2 and anti-HIV-1 group O antibodies [12].

The five rapid HIV tests approved in Europe for use on FSB or OF were performed in the following order on samples from each subject: OraQuick Advance Rapid HIV 1/2 antibody test® (Orasure/Orgentec) was performed first on oral fluid (OF) and then on finger-stick whole blood (FSB), followed by the other four tests on FSB: Vikia HIV 1/2® (bioMérieux), Determine HIV 1–2® (Unipath, Inverness), Determine**®** HIV-1/2 Ag/Ab Combo® (Determine 4G for 4^th^ generation; Unipath, Inverness) and INSTI HIV-1/HIV-2® (Biolytical, Nephrotek). The particularity of the Determine**®** HIV-1/2 Ag/Ab Combo® test is that it detects both P24 antigen and anti-HIV antibodies and can therefore potentially reduce the window of seronegativity during primary infection [13]. The same batch of tests was used throughout the study. The characteristics of the rapid tests are summarized in Table 1.

**Table 1:** Technical characteristic of EU-approved HIV screening rapid tests for use on whole blood and/or oral fluid.

Test	Manufacturer	Principle and antigens coated on membrane solid phase	Binding revelation reagents	Procedural Control coated on solid phase	Volume Time for reading
Oraquick ADVANCE Rapid HIV-1/2 Antibody test	Orasure technologies (USA)	Immunochromatography HIV1 group M – group O (gp41) and HIV2 (gp36) synthetic peptides	Protein A labelled with reddish purple	Goat anti human Ig G	5 µL blood oral fluid cravicular collection 20–40 mn
VIKIA HIV 1/2	bioMérieux (France)	Immunochromatography HIV1 group M - group O (gp41) and HIV2 (gp36) synthetic peptides	Antigens linked to blue colored microspheres	Colored bovine serum albumin	75 µL 20–30 mn
Determine HIV 1–2	Orgenics Ltd (Israel)	Immunochromatography HIV1 (gp41) and HIV2 (gp36) recombinant proteins synthetic peptides	Antigens linked to colloidal selenium	Anti HIV antibodies HIV peptide	50 µL 15–60 mn
INSTI HIV ½	Biolytical (Canada)	Immunofiltration HIV1 (gp41) and HIV2 (gp36) recombinant proteins	Protein A labelled with blue indigo	Protein A	50 µL5 mn
Determine Combo Ag AC HIV 1–2	Orgenics Ltd (Israel)	Immunochromatography 1/HIV1 (gp41) and HIV2 (gp36) recombinant proteins synthetic peptides 2/Avidine to capture anti p24 labelled antibodies	Antigens linked to colloidal selenium Anti HIV-1 p24 antibodies linked to biotin	No data available	50 µL 15–60 mn

The tests were done by two physicians and two technicians specially trained for the study, in keeping with the manufacturers' recommendations. Oral fluid was obtained with a swab, between the upper and lower teeth and gums, following the manufacturers' instructions. Finger-stick whole blood was obtained with a microlancet after hand warming. In case of insufficient sample volume, patients underwent a second finger-stick. Blood was collected with a capillary tube and immediately deposited on the different test strips, as recommended. After 20 minutes (except for the INSTI test, which is read immediately), all the tests were read by a single investigator, different from the one who performed the tests. Consequently, the reader was unaware of the subjects' HIV serostatus between HIV-infected patients and healthy volunteers.

Venous blood was drawn at the same time and serum was isolated by centrifugation (10 min, 3000 *g*) and stored at −20°C until use. The results were recorded as positive, weakly positive (faint band), negative or invalid (non reactive internal control). In case of false-negative and/or invalid results, the patient's serum sample was thawed and retested with the corresponding falsely negative or invalid rapid test(s) and EIA. A fourth-generation EIA, the Architect® i2000SR Abbott HIV1/2 assay was considered the gold standard because of its high sensitivity for early seroconversion [14]. Architect® i2000SR assays were performed on stored frozen samples. P24 HIV-1 antigen, when present, was quantified with the Vidas HIV-1 p24 Antigen assay (bioMérieux, Marcy l'Etoile, France).

### Statistical analysis

The sensitivity of the different tests was defined as the number of positive and weakly positive tests divided by the number of valid tests. Sensitivity on the 6 different tests was compared using a logistic regression model using generalized estimating equations (GEEs) approach to take into account correlated data [15]. The Mc Nemar test for paired samples was used to compare the sensitivities of the OraQuick test on OF versus FSB [16]. The same statistical test was used to compare the sensitivity of the different rapid tests on FSB, and to compare the sensitivity of these tests on either FSB or serum. Indeed, rapid tests which were falsely negative on FSB were repeated on the corresponding sera. We did not repeat all the tests on serum, assuming that tests positive on FSB would also be positive on serum.

Baseline characteristics of the patients in whom all the tests were positive were compared with those of patients with at least one negative test, using the chi-square test and Wilcoxon rank sum test in order to identify factors associated with false-negative results. A p value of <0.05 was considered to denote statistical significance. The SAS 9.1 software package (SAS Inc, Cary, NC) was used for all analyses.

## Results

The HIV-infected patients were mostly men (83%) of European origin (59.8%), with HIV-1 subtype B infection (63.3%), under antiretroviral therapy (68.5%), and with plasma HIV-1 RNA <200 cp/ml (57.8%). Their median CD4 cell count was 437/mm^3^. Patient acceptance of this protocol was particularly high, as recruitment took place during quarterly follow-up visits by the patients' usual doctors.

Sensitivity was 86.5% [81–90.5] with OraQuick OF, 94.5% [90.4–96.9] with OraQuick FSB, 98.5% [95.6–9.5] with Vikia, 94.9% [90.8–97.2] with Determine, 95.8% [91.6–97.9]] with Determine 4G and 99% [96.3–99.7 with INSTI (p<0.0001). OraQuick was significantly less sensitive on OF than on FSB (p = 0.008). The sensitivity of OraQuick on OF was also significantly lower than the sensitivity of the tests using FSB (p = 0.0002, 0.006, 0.0002, and 0.004 for Vikia, Determine, INSTI and Determine 4G, respectively). The sensitivity of OraQuick and Determine on FSB was significantly lower than that of INSTI (p = 0.025 and 0.03, respectively).

Overall, 60 tests (5.2%) were falsely negative on samples from 36 patients. Among the six patients with three or more negative tests, two had recent HIV infection, and four had undetectable plasma viral load on antiretroviral therapy; one of the latter patients was infected by HIV-1 group O. The 2 seroconventer patients were infected by HIV-1 subtypes B and F, less than 2 months previously. Recent infection was confirmed by a weakly positive 4^th^ generation EIA test and by Western blot profiles showing a typical seroconversion pattern with isolated Gag-Env weak reactivity in both subjects without Pol p31 band [17]. Western blot follow up confirmed the seroconversion in both cases. Their viral loads were 5 206 179 and 14 836 copies/ml, respectively. P24 antigen was detectable at 380 pg/ml in the plasma of the first patient but was not detectable in the other patient. All rapid tests were negative in both patients, with the exception of the INSTI test, which was weakly positive in the latter patient. No P24 antigen band was seen in the Determine 4G test in either of the patients with recent infection.

Four other patients had three or more negative rapid tests:

- Only the Vikia and INSTI tests were positive in a 34-year-old man with HIV-1 group O infection. Viral load was undetectable on HAART and his CD4 cell count was 159/mm3. When tested on serum, only OraQuick remained negative.
- Only the Vikia and INSTI rapid tests were positive (weak reactivity) in a 42-year-old Caucasian man who was diagnosed with HIV-1 B subtype infection in March 2005. His viral load was undetectable on HAART and his CD4 cell count was 675/mm^3^. When tested on serum, OraQuick remained negative.
- OraQuick (OF and FSB) Vikia, Determine and Determine 4G were negative in a 40-year-old Caucasian man diagnosed with HIV-1 subtype B infection in March 1999. Viral load was undetectable on HAART and his CD4 cell count was 861/mm3. When tested on serum, only OraQuick remained negative.
- The OraQuick (OF and FSB) and INSTI tests were negative in a 27-year-old woman of African origin who had been diagnosed with HIV infection (indeterminate subtype) in September 2004. Her viral load was undetectable on HAART and her CD4 cell count was 831/mm3. INSTI remained negative on serum and OraQuick was weakly positive.

Among the six patients with HIV-2 infection, two had a false-negative OraQuick test on OF but both were positive on FSB. These two patients were receiving antiretroviral therapy and their plasma viral load was undetectable in an HIV-2-specific assay. All 20 HIV-negative controls were negative in all the rapid tests. Of note, 39 (3.2%) tests were invalid, owing the absence of the control line. The Determine 4G test gave 33 invalid results (16.5%) (Table 2).

**Table 2:** Sensitivity of five rapid HIV tests in 200 HIV-infected patients, using either oral fluid (OF) or finger-stick whole blood (FSB).

	Oraquik OF	Oraquick FSB	Vikia FSB	Determine FSB	INSTI FSB	Determine 4G FSB
**Invalid test**	0	0	0	4	2	33
**Negative test**	27	11	3	10	2	7
**Weakly positive test** *	10	6	1	1	4	7
**Positive test**	163	183	196	185	192	153
**Overall sensitivity** **% of valid tests** **[95% CI]**	**86.5% [81–90.5]**	**94.5%** **[90.4–96.9]**	**98.5%** **[95.6–99.5]**	**94.9%** **[90.8–97.2]**	**99%** **[96.3–99.7]**	**95.8%** **[91.6–97.9]**

Sensitivity was calculated by dividing the sum of positive and weakly positive tests by the number of valid tests. Tests without a visible control line were considered invalid.

*only a faint band was visible, but the test was considered positive.

Among the 18 patients (33 tests) with at least one negative FSB test, all but six had positive results on serum (5 negative with OraQuick previously falsely negative in FSB and 1 with INSTI) (Table 3). These 6 patients with negative rapid tests on serum were the same as those with at least three negative tests on FSB (see above), i.e. the two patients with recent HIV-infection, and four patients with undetectable plasma viral load on antiretroviral therapy. Assuming that the tests positive on FSB would also have been positive on serum, the sensitivities of OraQuick, Determine and Determine 4G were significantly better on serum than on FSB with 94.5% [90.4–96.9] vs 97.5% [94.2–98.9] p = 0.04, 94.9% [90.8–97.2] vs 100% [98.1–100], p = 0.004, and 95.8% [91.6–97.9] vs 100% [98–100], p = 0.02, respectively (Table 3). When the 39 tests with invalid results on FSB were repeated on serum, only four remained invalid (all with the Determine 4G).

**Table 3:** Sensitivity of five rapid HIV tests in 200 HIV-infected patients, combining the results for finger-stick whole blood and, when the latter was negative, for serum.

	Oraquick FSB	Vikia FSB	Determine FSB	INSTI FSB	Determine 4G FSB
**Positive test in serum**	**6/11**	**3/3**	**10/10**	**1/2**	**7/7**
**Overall serum sensitivity % [95% CI]**	**97.5% [94.2–98.9]**	**100% [98.1–100]**	**100% [98.1–100]**	**99.5% [97.2–99.9]**	**100% [98–100]**
**P**	**0.04**	**0.25**	**0.004**	**1**	**0.02**

Differences in sensitivity between whole blood and serum were analyzed with the Mc Nemar test for paired samples.

The P24 band of the Determine 4G test was never positive on FSB, even in the patient with 380 pg P24/ml of serum. Determine 4G was also negative for P24 antigen on serum from this patient.

Only plasma HIV-1 RNA level below 200 copies/ml and elevated CD4 cell counts were significantly associated with the risk of having at least one negative test. However, the significant association (p = 0.04) with the CD4 cell count disappeared in multivariate analysis and only plasma HIV RNA <200 cp/ml remained significantly associated with the risk of having at least one false-negative result (odds ratio: 3.67, 95% CI: 1.52–8.84, p = 0.009) (Table 4).

**Table 4:** Comparison of patient characteristics according to HIV screening rapid tests results on whole blood and/or oral fluid results.

	All tests positive	≥1 negative test	p-value uni-variate analysis	p-value multi- variate analysis
	n = 164	n = 36		
Median age (years)	41	44.5	0.24	
Female (n, %)	28 (17%)	6 (16.6%)	1.00	
CDC stage C (n,%)	37 (22.5%)	11(30.5%)	0.39	
Caucasian	93 (57.1%)	26 (72.2%)	0.24	
Sub-Saharan African	53 (32.5%)	8 (22.2%)		
Other	17 (10.4%)	2 (5.6%)		
HBV or HCV infection (n,%)	16 (9.8%)	3 (8.3%)	1.00	
ARV therapy (n,%)	108 (65%)	29 (80.5%)	0.11	
Date of HIV infection ≤2002	96 (58.5%)	19 (52.8%)	0.58	
Median CD4 cell count	416	500	0.04	0.11
HIV VL <200 cp/ml (n,%)	87 (53.1%)	29 (80.5%)	0.004	0.009
HIV-1 B subtype (n,%)	106 (64.6%)	20 (55.6%)	0.34	
HIV-2	4 (2.4%)	2 (5.5%)		
HIV-O		1 (2.5%)		
others		2 recent infection		

*HIV genetic diversity* in our population was high, with 36.7% of non B subtypes and a large panel of complex recombinant strains. Six patients were also infected by HIV-2 and one by HIV-1 group O. Table 5 summarizes the results according to the type and subtype. There was no significant difference in sensitivity among the different rapid tests for B or non B HIV-1 subtype infection (Fischer exact test, p>0.25).

**Table 5:** Sensitivity of rapid test detection according to the HIV genotype.

	N 199*		Oraquick OF	Oraquick FSB	Vikia FSB	Determine FSB	Insti FSB	Determine 4G FSB
HIV-1 Subtype B	126	Positive (%)	**111** (88)	**119** (94)	**124** (97)	**118** (94)	**123** (97)	**96** (76)
		Negative	**15**	**7**	**2**	**5**	**1**	**5**
		Invalid	-	-	-	**3**	**2**	**25**
HIV-1 Subtype Non B**	58	Positive (%)	**51** (87)	**55** (94)	**57** (98)	**52** (89)	**58** (100)	**49** (84)
		Negative	**7**	**3**	**1**	**5**	-	**2**
		Invalid	**-**	**-**	**-**	**1**	**-**	**7**
HIV-2	6	Positive	**4**	**6**	**6**	**6**	**6**	**6**
		Negative	**2**	**-**	**-**	**-**	**-**	**-**
not typable	9	Positive	**7**	**8**	**9**	**9**	**8**	**8**
		Negative	**2**	**1**	**-**	**-**	**1**	**-**
		Invalid	**-**	**-**	**-**	**-**	**-**	**1**

*Failure from PCR, serotyping insufficient volume in one sample;

**HIV-1 Subtype non B: A (3); D (2); F (2); J (1); O (1); CRF01 (3); CRF02 (17); CRF06 (1); CRF19 (1); Recombinant B/CRF02 (1); Complex recombinant (2); Serotyped as non B (24). Fischer exact test, p>0.25.

## Discussion

This is the first study to compare the sensitivity of EU-approved rapid HIV screening tests, on oral fluid, capillary blood and serum. Among the 200 HIV-infected volunteers included in this study, rapid test sensitivity ranged from 86.5% [81–90.5] to 99% [96.3–99.7]. Thirty-six patients had a false-negative result in at least one of the six tests. OraQuick was the least sensitive test on both OF and whole blood and also yielded the largest number of “weakly positive” results. OraQuick sensitivity improved from 86.5% to 94.5% (p = 0.008) when FSB rather than OF was used, and to 97.5% when serum was used (p = 0.04, Table 3). On testing serum from patients with false-negative tests on FSB, only six tests remained falsely negative (OraQuick in 5 cases, INSTI in one case). These rapid tests are usually used to screen patients exposed to or suspected of being infected by HIV. In this indication, it is recommended to use two tests simultaneously to improve sensitivity [18], [19]. In our population, most of the patients were already treated and 29 (80.5%) of the 36 patients with at least one negative rapid test had undetectable viral load. This relation with undetectable viral load was significant in both univariate (p = 0.004) and multivariate analysis (p = 0.009) (Table 4).

In a study of 327 patients, Delaney found more false-negative results with OraQuick on OF than on whole blood (99.1% versus 99.7%) [20]. Likewise, in a study of 81 patients in South Africa, the sensitivity of OraQuick was only 96.3% on OF, compared to 100% on whole blood [21]. In a study of 139 patients, the sensitivity of OraQuick was lower on OF (97.8%, with 3 false-negatives) than on serum or plasma (100%) [22]. In these studies, all the patients with false-negative OraQuick results had undetectable viral load on antiretroviral therapy. The relation between falsely negative oral fluid rapid tests and antiretroviral therapy is well established and explains why in our study, with a large proportion of patients on antiretroviral therapy, we obtained a sensitivity of only 86.5% [81–90.5] with OraQuick on OF. This lower sensitivity on oral fluid from treated patients has previously been reported, but not in a study comparing different assays simultaneously. Some of the rapid test evaluated here were reactive with a large majority of the samples, whatever the patients' status. Lower reactivity due to treatment was a problem with some assays but not with others. The lower sensitivity of OF tests in patients on treatment is mentioned in the package inserts. Physicians must be aware of this limitation, as some such patients (if unconscious, for example) may be unable to state their HIV serostatus.

The negativity of rapid tests on whole blood from the two patients with recent infection confirms their lack of sensitivity during this period. However, except for OraQuick, all the tests were reactive on serum from both patients. These patients had specific antibodies on Western blot (WB) and one already had undetectable P24 antigenemia, indicating that they were in the later stages of primary infection. Evaluation of rapid tests for diagnosis of recent HIV infection in healthcare settings is hindered by the difficulty of recruiting such patients, but the lack of rapid test sensitivity in this setting has already been underlined. In 2007, Stekler *et al* reported three cases of recent HIV infection (less than 6 months) with negative OraQuick results on whole blood [23]. In 2009, the same authors reported the limits of OraQuick rapid testing on OF and FSB in the USA [24]. Rapid testing was positive in 153 (91%) of 169 HIV-infected men who have sex with men, all of whom were positive by EIA and/or nucleic acid testing. Fourteen patients with primary infection and one profoundly immunodepressed patient were positive in a 4^th^ generation EIA test detecting both P24 antigen and anti-HIV antibodies [24]. Such combined assays are highly sensitive, detecting less than 15 picograms of P24/ml [25]. P24 antigen detection is useful for closing the primary infection “window” period [17]. Determine 4G, the first such rapid test, needs to be made more sensitive, as none of the whole-blood samples from our 200 patients reacted with the P24 antigen line. This could be due to the relatively early stage of HIV infection and to the control of viral replication by treatment or to the African origin of many of our patients (61 cases). Similarly, Tardy *et al*, using serum samples from HIV-infected patients positive for P24 antigen, found that none of the 17 patients with less than 50 pg of P24 antigen per milliliter of serum was positive for P24 in the Determine 4G test, while only 4 of the 9 patients with values between 50 and 400 pg/ml were positive [26]. There is insufficiency of literature on rapid test in health care setting with recent HIV infection due to the difficulty to include such patients [23], [24], [27]. This lower sensitivity of rapid tests during primary HIV infection increases the risk of misdiagnosis in patients with active viral replication and a high risk of transmission, implying that these tests should be used with care, particularly on OF or whole blood, in populations with a high incidence of HIV infection, especially in primary care or emergency settings [28]. Possible explanations for this lower sensitivity of rapid tests include weaker antibody affinity due to hemolysis, dilution in whole blood, the short migration or filtration time for antigen-antibody binding, and reaction at room temperature instead of 37°C as in EIA tests [29].

HIV antigenic diversity has been implicated in poor antibody detection by rapid tests, particularly in case of variant or primary infection [8], [27]. In our study, the difference in the frequency of false-negative results for subtype B and non B HIV-1 group M was not statistically significant. HIV screening tests use synthetic antigens based on sequences of HIV-1 subtype B viruses that circulate in western countries. In case of HIV-1 non B or highly divergent HIV infection, non-B antibodies binding affinity could be limited, particularly during the seroconversion phase [30]. HIV-2 infection was detected by all the tests used here, except for OraQuick oral fluid, which missed two cases. A patient infected by HIV-1 group O was negative in the OraQuick blood and OF tests and also in both Determine tests. The poor performance of the rapid tests for the patient infected by HIV-1 group O in this study confirms the difficulty of recognizing this rare variant [31]. It is noteworthy that HIV-2 and HIV-O account for 2% of all new infections each year in France [32]. In addition, rapid tests are widely used in sub-Saharan Africa, where such viruses circulate at higher levels.

Post-marketing studies of the OraQuick test on whole blood and oral fluid showed a lack of specificity, with positive predictive values of 90.00% (range: 50.00–100%) for OF and 99.24% (range: 66.67–100%) for FSB [33]. In a more recent study, the specificity of OraQuick on OF declined significantly as the end of the shelf life approached [34]. Owing to the design of this study, focusing only on sensitivity in patients with known infection and using only a small number of HIV-negative patients to blind the reading procedure, we are unable to evaluate positive or negative predictive values. The accuracy of diagnostic tests depends on the prevalence of a disease in the population. This is of particular importance in highly endemic countries where test positivity has a high probability of being truly positive. Whatever the test, the strategy and the clinical setting, quality control remains crucial [35] and these devices are not considered alone sufficient to confirm HIV infection. International guidelines recommend that, whatever the HIV prevalence, a positive rapid test must be confirmed by EIA or at least one other rapid test [18]. WHO recommends using a first assay with 100% sensitivity and good specificity [18]. In case of positivity, the confirmatory assay should have 100% specificity and good sensitivity. This strategy is not easy in practice, and a recent study performed in Africa using current diagnostic tests and recommended algorithms clearly underlined the need for well-evaluated and high-quality rapid tests [36].

All the tests were easy to interpret, including the internal procedural control, with the exception of the 4th generation Determine test. The high rate of invalid 4th generation Determine results (16%), due to the lack of control band reactivity, could be due to high antibody titers consuming the conjugate at the test line and/or to non specific slower migration with whole blood and/or avidin-biotin-anti-P24 antibodies competing with the reagent included in the strip and patient antibodies. Alternatively, the low anti-HIV antibody titers in our treated population could interfere with binding to the control band. This phenomenon was less frequent with the corresponding serum samples, showing that the use of whole blood hinders sample migration and/or antigen-antibody binding on the test strip. Unreliable internal controls are a major drawback. Rapid tests usually have neutral migration controls rather than the positive and negative reaction controls used in EIA methods. This lack of proper controls is a further drawback.

Our study has several limitations. In particular, its size was somewhat limited and the results we obtained are potentially only applicable to the population studied, i.e. mostly patients infected with HIV-1 subtype B, having undetectable plasma HIV RNA levels and high CD4 cell counts on antiretroviral therapy. We were only able to enroll two patients with recent HIV infection. Also, owing to the study design, with five tests performed simultaneously in the same order, technical problems such as insufficient OF or blood volume for some tests may have affected the results. However, we took these issues into account, notably by repeating the finger-stick procedure if necessary. The sensitivity results were not influenced by the order in which the tests were performed. For example, INSTI was the last test but had one of the highest sensitivities (99%). Finally, we compared the sensitivity of FSB with that of serum, although we repeated only negative FSB tests, assuming that patients with positive FSB results would also be positive on serum. The need for fresh blood samples collected at a point of care hinders repeatability studies but, as shown by the overlapping confidence limits of the different tests, the statistically significant differences in sensitivity might not be confirmed if the study were to be repeated.

In conclusion, this first study comparing different EU-approved HIV rapid tests in a healthcare setting confirms that the such tests should be used with caution on whole blood and OF, especially in patients with recent infection and in patients on antiretroviral therapy. The tests all tended to perform better on serum than on finger-stick blood or oral fluid. Given their potentially important public health implications, these limitations need to be confirmed in further real-time studies.
